# Bladder Sensation and Gait: A Cross-Sectional Observational Study of Dual-Task Interference in Older Women

**DOI:** 10.1007/s00192-026-06573-z

**Published:** 2026-03-30

**Authors:** Daniela Aldabe, Fernanda Kruger Romio, Ailish Sullivan, Felipe de Souza Stigger

**Affiliations:** 1 School of Physiotherapy, University of Otago, Dunedin, New Zealand; 2 Curtin School of Allied Health, Faculty of Health Sciences, Curtin University, Perth, Australia; 3 Physiotherapy Undergraduate Program, Federal University of Health Sciences of Porto Alegre, Porto Alegre, Brazil; 4 Department of Physiotherapy, Federal University of Health Sciences of Porto Alegre, 245 Sarmento Leite St, Porto Alegre, Rio Grande Do Sul 90050-170 Brazil; 5 Curtin University, Kent St, Bentley, WA 6102 Australia

**Keywords:** Bladder Control, Cognitive Aging, Falls, Gait, Urinary Incontinence

## Abstract

**Introduction and Hypothesis:**

Older women with urinary incontinence (UI) have an elevated risk of falling, a phenomenon that may be partly explained by the attentional demands required to manage bladder sensations during mobility. The objective of this study is to determine whether the urge to urinate in older community-dwelling women presenting with a range of UI severity, influences dual-task performance.

**Methods:**

This cross-sectional observational study included women aged ≥ 65 years who completed the Timed Up-and-Go (TUG) test under single-task conditions. They also completed a motor task (coin transfer) and a cognitive task (phonetic verbal fluency) under dual-task conditions. All tasks were performed with no desire to void and desire to void. Urinary incontinence severity was measured using the International Consultation on Incontinence Questionnaire–Short Form (ICIQ-SF). Repeated measures analysis of variance (ANOVA) compared dual-task effects across bladder conditions. Kendall’s correlation examined associations between UI severity and TUG test performance. Forty-two women (72.90 ± 5.81 years) with varying degrees of UI participated.

**Results:**

The desire-to-void condition significantly impaired gait performance. Dual-task effects were greater during the desire-to-void state than during the no-desire-to-void state across motor and cognitive tasks (*p* < 0.01). Neither TUG test performance nor dual-task effects correlated significantly with ICIQ-SF scores. These results indicate that bladder sensation, rather than UI severity, contributes to performance differences.

**Conclusions:**

The desire to void adversely affects dual-task gait performance in older women, regardless of UI severity. Bladder-related attentional demands may impair functional mobility and should be considered during fall-risk assessment and intervention planning for older adults.

**Supplementary Information:**

The online version contains supplementary material available at 10.1007/s00192-026-06573-z

## Introduction

Falls and urinary incontinence (UI) are among the most common geriatric syndromes [1], with both conditions affecting over 30% of community-dwelling older women [2, 3]. Older women with UI face a higher risk of falls than those without UI [4]. The underlying mechanisms behind the association of UI and falls are not yet fully understood. A plausible explanation is that the increased desire to void requires higher cognitive focus on bladder control, diverting attention from safe walking [5]. Evidence demonstrates that older women decrease gait velocity and stride length and increase gait variability when experiencing a strong desire to void [6]. Also, the severity of urgency and mixed UI correlated with altered gait parameters [6]. In addition, older adults with overactive bladder who wet when experiencing urinary urgency present similar gait alterations to those caused by a distracting cognitive task [7].

Dual-task walking is critical to safety and functional independence [8]. In daily life, secondary tasks create demanding situations, such as walking to the toilet while simultaneously navigating environmental hazards and managing urgency symptoms. Understanding the impact of secondary cognitive and/or motor tasks while experiencing a desire to void is crucial. Although previous studies have explored bladder-related sensations and cognitive distraction during walking, to our knowledge no study has investigated how experimentally induced desire to void modulates dual-task interference during gait using both motor and cognitive secondary tasks in older adults with UI. Hence, this study is aimed at:

1. Exploring the effects of two attention-demanding tasks on gait performance in older women
2. Establishing whether a moderate desire to void influences dual-task walking performance
3. Correlating UI severity with walking performance and dual-task effects
4. Describing dual-task interference pattern on gait and secondary tasks for older women with continence and incontinence

We hypothesized that divided attention during dual-tasking will negatively impact gait performance. Also, altered attention demand whilst attempting dual-task walking and maintaining continence may require more significant use of cognitive resources impacting gait. Finally, we expect to observe a positive correlation between UI severity and dual-task effects during walking while experiencing a desire to void.

## Materials and Methods

### Study Design

This is a cross-sectional, repeated-measures, laboratory-based study composed of community-living older women. This study was approved by the Research Ethics Committees from the University of Otago and the Federal University of Health Sciences of Porto Alegre (H21/150 and 27093419.7.0000.5345, respectively).

### Participants

Participants were recruited by public advertisements in the community and on social media between December 2021 and March 2023. Recruitment took place in two different countries, New Zealand and Brazil. Participants were included if they were aged 65 years and over; community-living, independent in activities of daily living; could walk without assistance; able to provide self-reported data; and could understand and sign informed consent. Participants were excluded if they presented musculoskeletal, metabolic, infection or cardiac disorders or visual or hearing impairments that could interfere with the experiment.

### Data Collection

Data collection took place in two different institutions: University of Otago, in New Zealand and Federal University of Health Sciences of Porto Alegre, in Brazil. After confirming eligibility, all participants provided informed consent following the Declaration of Helsinki. Next, participants completed a short sociodemographic questionnaire and questionnaires on continence status/severity (International Consultation on Incontinence Questionnaire—Short Form [ICIQ-SF]) [9, 10], cognition (The Montreal Cognitive Assessment [MoCA]) [11, 12], and balance confidence (Balance Confidence Scale [ABC-16]) [13, 14]. Afterwards, participants went to either the Human Movement Laboratory of the Federal University of Health Sciences of Porto Alegre or the School of Physiotherapy Exercise Gym Facility of the University of Otago to perform the dualtask data collection protocol.

### Dual-Task Data-Collection Protocol

The dual-task paradigm adopted in this study consisted of executing the primary walking task performed separately and with a secondary motor task and cognitive task. As the primary walking task, participants were asked to perform the timed up-and-go (TUG) test [15, 16]. The variable of interest was the time to complete the TUG test in seconds. The coin transference task (CTT) was chosen as a secondary motor task [17]. For the CTT, participants transferred ten coins of 50 cents one at a time, with the dominant hand, from the pocket on the nondominant side to the pocket on the dominant side using a specific apron. The parameter of interest was the coin transference rate (coins/min). The cognitive secondary task included the phonetic verbal fluency (PVF) task. For the PVF task, participants were asked to recall as many words as possible, beginning with the letters F, A or S, for 1 min [18]. The number of appropriate words generated in 1 min was considered. To minimize learning effects, participants underwent a brief familiarization procedure before data collection. Participants practiced the TUG test, CTT, and PVF tasks separately to ensure understanding of the instructions and safe execution, but no extended dual-task training was provided.

### Desire-to-Void Conditions

Participants performed the dual-task protocol followed by two conditions: no desire to void and moderate desire to void. To achieve a moderate desire to void, participants were given 500 ml of water to drink and then waited until they experienced a moderate desire to void. Moderate desire to void was determined using the Urinary Sensation Scale, a five-point validated scale developed to assess feelings of urinary desire to void [19]. A score of 3 out of 5 (enough desire to void discomfort, need to stop the usual activity or task, and go immediately to the bathroom) was considered.

Participants performed the five attempts consecutively, and no standardized rest interval was imposed between trials. The only pause between attempts occurred because of the pseudo-randomized task order and during the transition to the moderate-desire-to-void condition, when participants waited until they self-reported moderate urgency following water intake. This waiting period was not standardized because the time required to reach moderate urinary urgency is individual and subjectively determined based on the urinary sensation scale. Pseudo-randomization was implemented by generating a list of possible assessment sequences based on the final target sample size. Each participant was then individually assigned to one sequence using a simple random draw, which determined whether the protocol began under the empty bladder or moderate-desire-to-void condition. Within each bladder condition, the order of single-task and dual-task trials was also randomly assigned.

### Outcome Measures

Primary outcome measures included time to perform the TUG test and dual-task effects (DTEs). DTEs on TUG test performance was defined as a percentage change of time needed to conclude the test compared with the single-task condition and was calculated as follows: (([single-task value—dual-task value]/single-task value) × 100%) [20]. Thus, a negative value indicates that TUG test performance was worse than the TUG test in the dual-task condition compared with the single-task condition. Secondary outcome measures considered for this study included the ICIQ-SF, MoCA and ABC scores.

### Sample-Size Determination

Sample size was estimated based on a moderate effect size for the DTE (Cohen’s dz = 0.55) reported in a similar dual-task walking paradigm. We used a two-tailed matched-pairs model, with alpha set at 0.05 and power set at 0.80. Based on this, the minimum required sample was 28 participants [21].

### Statistical Analyses

Age, MoCA score, TUG test performance, and DTEs were presented as means and standard deviations. Data normality was verified using the Shapiro–Wilk test. TUG test performance and DTEs during different experimental conditions were analyzed following a repeated measures analysis of variance (ANOVA) followed by the Bonferroni post hoc test. A subgroup analysis between participants with continence and those with incontinence, and between participants with MoCA < 26 and ≥ 26, were also performed. For all comparisons, alpha was set at 0.05. Kendall correlation was used to assess the correlation between ICIQ-SF and DTE. All data analyses were performed using SPSS (version 22).

To explore the interaction between motor and cognitive DTE during different void conditions and to understand the attentional strategy adopted by participants during dual-tasking, the conceptual framework proposed by Plummer et al. was used [22]. Thus, operating characteristic graphs were created and analyzed descriptively.

## Results

### Participant Characteristics

From the 44 older women assessed for eligibility, 2 did not meet inclusion criteria. Forty-two older women (72.90 ± 5.81 years) participated in this study and were included in the data analysis. The MoCA scores show that 23 participants met the threshold for normal cognition (25.00 ± 5.54). Although 19 participants scored below 26 points, they were all community-living, independent in their activities of daily living, thus meeting the inclusion criteria of the present study. According to the ABC test, most participants presented high or moderate physical functioning. Participants’ ICIQ-SF scores ranged from no incontinence to severe incontinence. However, most had slight or moderate incontinence (Table 1).

**Table 1:** Characteristics of the study population

Characteristics	Sample (*N* = 42)
Age in years, mean (SD)	72.9 (5.8)
MoCA Score, mean (SD)	24.0 (5.5)
No. participants < 26	19
No. participants ≥ 26	23
ABC Score, mean (SD)	81.1 (18.8)
No. participants 81–100%	26
No. participants 51–80%	12
No. participants < 50%	4
ICIQ-SF Score, mean (SD)	5.4 (5.2)
No. participants 0 (none)	13
No. participants 1–5 (slight)	10
No. participants 6–12 (moderate)	13
No. participants 13–18 (severe)	6
No. participants 19–21 (very severe)	0
Type of incontinence as per ICIQ-SF	
No of continent participants	13
No. of participants with urgency incontinence	15
No. of participants with stress incontinence	7
No. of participants with mixed incontinence	7

*ABC* Activities-Specific Balance Confidence Scale, *ICIQ-SF* International Consultation on Incontinence Questionnaire–Urinary Incontinence Short Form, *MoCA* Montreal Cognitive Assessment, *SD* standard deviation

### Walking Performance

**Fig. 1:**
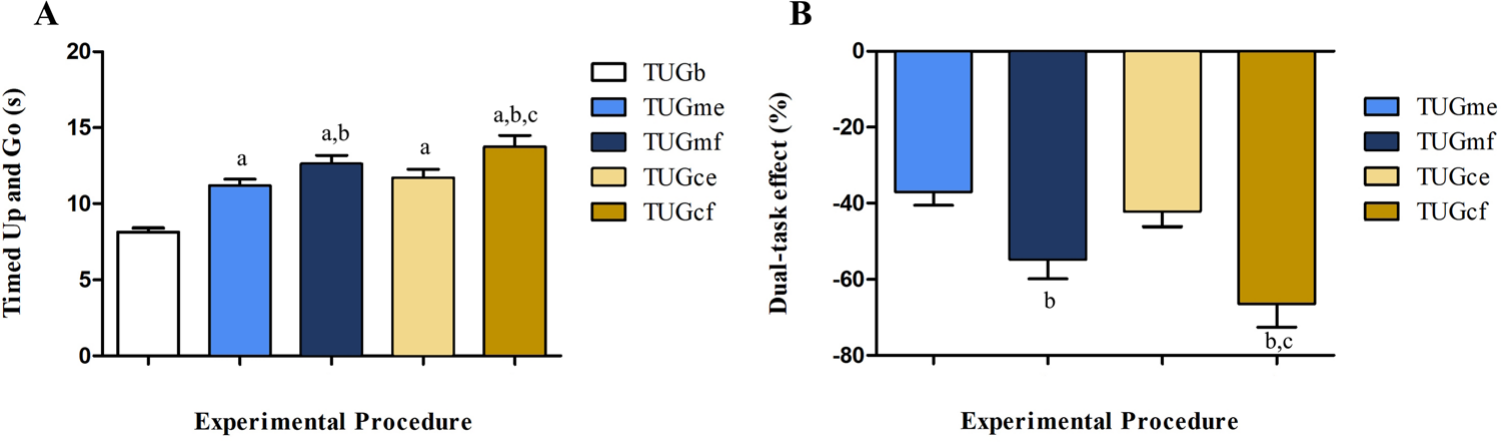
Impact of all experimental conditions on the walking task. **A** timed up-and-go (TUG) test performance and **B** dual-task effect of participants under five experimental conditions: *TUGb* TUG test baseline, *TUGme* TUG test motor dual-task empty bladder, *TUGmf* TUG test motor dual-task full bladder, *TUGce* TUG test cognitive dual-task empty bladder, *TUGcf* TUG test cognitive dual-task full bladder. *a* Significantly different from single-task performance, *p* < 0.001. *b* Significantly different from respective empty-bladder condition, *p* < 0.01. *c* Significantly different from motor empty-bladder condition, *p* < 0.001

Absolute values of TUG test performance and DTEs during all experimental conditions are shown in Table 2 and Fig. 1. Repeated measures ANOVA demonstrated a significant effect of the experimental conditions on TUG test performance ([F(4,164) = 41,93; *p* < 0.001]). Post hoc comparisons showed that the time to perform the TUG test significantly increased when performing a motor (MD = −3.01, 95% CI [−3.88, −2.13], *p* < 0.001) or cognitive (MD = −3.53, 95% CI [−5.74, −3.17], *p* < 0.001) secondary task compared with single-task performance. Post hoc comparisons also indicated that experiencing a moderate desire to void significantly increased the time to perform the TUG test compared with the equivalent motor (MD = −1.44, 95% CI [−2.51, −0.37], *p* = 0.003) and cognitive (MD = −2.04, 95% CI [−3.38, −0.71], *p* < 0.001) no-desire-to-void condition. In addition, post hoc comparisons also demonstrated that the TUG test performance was significantly higher during cognitive dual-tasking with a moderate desire to void than during motor dual-tasking with no desire to void (MD = −2.56, 95% CI [−4.19, −0.93], *p* < 0.001).

**Table 2:** Dual-task interference on walking performance during empty and full-bladder conditions

Variable	Single-task performance	Dual-task performance*	
		Motor CT task	Cognitive PVF task
Empty bladder condition (no desire to void)	8.1 (1.6)		
TUG test (s), mean (SD)		11.1 (2.8)**	11.6 (3.6)**
DTE, mean (SD)		−37.0 (22.9)	−42.1 (26.1)
Full bladder condition (moderate desire to void)			
TUG test (s), mean (SD)		12.6 (5.5)**, ***	13.7 (4.8)**, ***, ****
DTE, mean (SD)		−54.7 (33.3)***	−66.3 (40.2)***, ****

*CT* coin transference task, *DTE* dual-task effect on timed up-and-go test time: [(single-task value—dual-task value)/single-task value] × 100, *PVF* phonetic verbal fluency, *SD* standard deviation

*A post hoc analysis was performed using the Bonferroni method

**Significantly different from single-task performance *p* < 0.001

***Significantly different from respective empty bladder condition *p* < 0.01

****Significantly different from motor empty bladder condition *p* < 0.001

Repeated measures ANOVA demonstrated a significant effect of the experimental conditions on DTE ([F(3,123) = 12,50; *p* < 0.001]). Post hoc comparisons indicated that moderate desire to void significantly elicited the greatest DTEs compared with the equivalent motor (MD = –17.67, 95% CI [−29.49, −5.84], *p* < 0.001) and cognitive (MD = −24.24, 95% CI [−38.67, −9.80], *p* < 0.001) no-desire-to-void condition. Also, post hoc comparisons demonstrated that the DTE was significantly higher during cognitive dual-tasking with moderate desire to void than during motor dual-tasking with no desire to void (MD = −29.32, 95% CI [−45.70, −12.94], *p* < 0.001).

Repeated measures ANOVA subgroup analysis demonstrated no interaction between experimental conditions and continence status (i.e., participants with continence versus those with incontinence; [F(4,160) = 0.27; *p* = 0.960]). No correlations were observed between the ICIQ-SF scores and time to perform the TUG test or DTEs during moderate desire to void (Table 3).

**Table 3:** Correlation between timed up-and-go (TUG) test performance and dual-task effects (DTEs) under empty- and full-bladder conditions and International Consultation on Incontinence Questionnaire–Short Form (ICIQ-SF) scores

Task	Condition			
	Empty-bladder condition (no desire to void)		Full-bladder condition (moderate desire to void)	
	|τb|	*p* value	|τb|	*p* value
TUG test baseline	0.180	0.111		
TUG test coin transference	0.154	0.174	0.177	0.116
DTE during coin transference	0.017	0.877	−0.066	0.558
TUG test phonetic verbal fluency	0.099	0.382	0.127	0.259
DTE during phonetic verbal fluency	0.024	0.834	−0.006	0.956

*|τb|* Kendall’s Tau B Kendall correlation: = 0.07 weak correlation, = 0.21 moderate correlation and = 0.35 strong correlation

*Significant correlation

On the other hand, repeated measures ANOVA subgroup analysis demonstrated an interaction between experimental conditions and cognitive status (i.e., participants with participants with MoCA scores < 26 and ≥ 26; [F(4,160) = 3.31; *p* = 0.039]). Post hoc comparisons showed that the time to perform the TUG test was significantly increased when performing the TUG test with a motor (MD = 3.21, 95% CI [1.18, 5.24], *p* = 0.003), cognitive (MD = 3.94, 95% CI [1.115, 6.78], *p* = 0.008) secondary task during moderate desire to void in participants with MoCA scores < 26 compared with participants with scores ≥ 26.

**Fig. 2:**
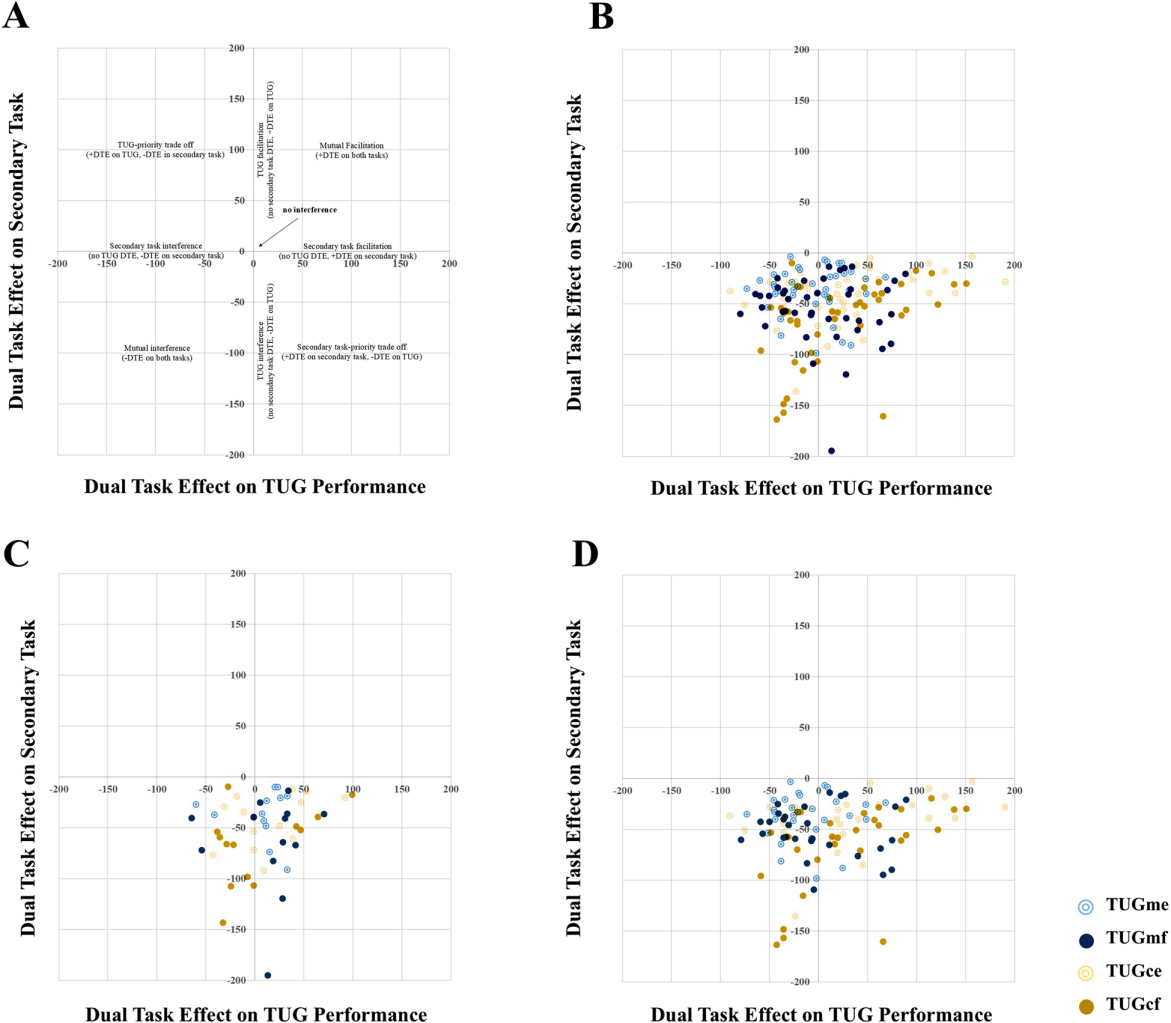
Patterns of dual-task interference on primarily TUG test performance and secondary task (motor and/or cognitive) during empty- and full-bladder conditions among participants. The scatter plot displays each individual’s dual-task effect (DTE) for the TUG task and for the secondary tasks (coin transference and phonetic verbal fluency (PVF) task, motor and cognitive respectively). DTE, calculated as [(single-task value—dual-task value)/single-task value] × 100%, equals zero when an individual’s performance on a given task is the same under the single-task and dual-task conditions. Positive DTE values indicate that performance was better under the dual-task condition relative to single-task performance (i.e., a higher number of pronounced words on the PVF task); negative DTE values indicate that performance deteriorated in the dual-task condition (i.e., increased time in TUG test performance). **A** Nine patterns of dual-task interference adapted from the conceptual framework of Plummer et al. [22]. **B** Performance operating characteristics of both patients with continence and those with incontinence. **C** Performance operating characteristics of participants with ICIQ Scores = 0 (i.e., participants with no signs of incontinence). **D** Performance operating characteristics of participants with ICIQ Scores ≥ 1 (i.e., participants with slight, moderate, and severe incontinence). Open symbols represent the empty-bladder condition, filled symbols represent the full-bladder condition

Figure 2 presents the operating characteristic graphs contrasting DTEs in primary and secondary tasks during both urgency conditions. Most participants had negative DTE values for the TUG task while presenting positive or negative DTE for the secondary coin transference and cognitive PVF task (Fig. 2b). Independent of the desire to void condition, most continent participants presented “TUG interference” or “secondary task-priority trade-off,” meaning that these participants had worse performance on the TUG task while maintaining (DTE no larger than 10%) or increasing their performance in the secondary tasks during the dual-task condition (Fig. 2c). Also, most participants with incontinence exhibited a “mutual interference” during the coin transference secondary task, meaning that these participants performed worse on both primary (TUG test) and secondary tasks in the dual-task condition. In contrast, most participants with incontinence tended to exhibit a “secondary task-priority trade-off” while performing a PVF secondary task. No participants exhibited “mutual facilitation,” “TUG facilitation,” or “TUG-priority trade-off.” Detailed operating characteristics of participants can be seen in Supplementary Fig. 1.

## Discussion

This study sought to establish whether a moderate desire to void impacts dual-task walking performance in healthy community-dwelling older women. We also examined whether self-reported UI severity influences walking performance and DTE during gait with a moderate desire to void. To our knowledge, this is the first study to systematically investigate dual-task gait performance under experimentally induced desire to void using both motor and cognitive secondary tasks. Our results show that

1. Decrements in walking performance are observed during dual-tasking
2. Moderate desire to void incited the greatest DTE, impacting walking performance compared with the equivalent motor and cognitive no-desire-to-void condition
3. No correlations between the ICIQ-SF scores and time to perform the TUG test or DTEs during both desire-to-void conditions were observed
4. Different performance operating characteristics can be observed comparing participants with continence and those with incontinence

In daily life, functional mobility involves walking while performing multiple tasks, enabling a response to environmental stimuli when walking. ​Hence, efficient and safe gait relies on cognitive processes [23]. Strong evidence demonstrates that when a simple motor task, such as walking, is coupled with a secondary task, the performance of one or both tasks can decrease [24]. This aligns with our results, demonstrating that community-dwelling older women have slower TUG test performance when dual-tasking.

It is important to note that, although dual-task conditions are typically associated with performance decrements, a substantial number of participants exhibited positive DTE values in the secondary tasks (coin transference and PVF), suggesting that dual-task costs were not uniformly expressed across domains. This pattern may indicate dual-task facilitation and/or individual task-prioritization strategies, whereby some participants allocated greater attentional resources to the secondary task while concurrently demonstrating a motor cost in the primary walking task (negative DTE in the TUG test). Such findings are consistent with a trade-off framework in dual-task performance, in which improvements in one domain may occur at the expense of another [25]. Moreover, dual-task models emphasize that allocation of attentional resources according to task prioritization can produce measurable performance trade-offs, and that trade-off and interference may coexist rather than being mutually exclusive. In addition, practice effects and increased arousal or engagement during dual-task execution may have contributed to improved secondary-task performance in some individuals [26]. Overall, these results highlight the heterogeneity of dual-task responses among older adults and reinforce the importance of interpreting motor and cognitive/manual outcomes simultaneously rather than in isolation.

Our results also show that the greatest impact on gait performance occurred during dual tasking while experiencing a moderate desire to void. There is limited evidence regarding the effects of bladder sensation on gait performance in older adults [6, 7]. However, the impact of desire to void on dual-task gait performance, particularly across both motor and cognitive dual-task paradigms, remains insufficiently investigated. As proposed by Booth et al. [27], maintaining bladder control during walking could be a source of divided attention. In the paradigm adopted in this study, we coupled the desire to void with a dual-task performance, which could be acknowledged as a “triple-task.” Bladder control requires a multidimensional process (cognition, emotion, self-reference, self-reflection, and deciding whether to void) in monitoring the external environment [28]. Thus, it is unsurprising that experiencing a moderate desire to void and the attention required to maintain continence and walking while dual-tasking decreased the resources attributed to gait control and adversely impacted walking ability.

Distinct neuropsychological theories on information processing could explain the decrements observed in dual-task performance. The capacity-sharing theory and bottleneck theory presume that the functional capacity of the human brain is limited [29–31]. In addition, the theory of multiple resources suggests that dual-task performance is likely to be hampered if two tasks share functional resources or common structures [32, 33]. Different mechanisms could explain the greatest DTE observed when participants performed a motor or cognitive dual-task while experiencing a desire to void. Recent data demonstrated that dual-tasking and experiencing a desire to void require similar functional networks. For instance, both situations have a greater prefrontal cortex and supplementary motor area activation [34–36]. Thus, combining a motor or cognitive dual-task with a desire to void (“triple-task”) could generate the most significant overlap activation pattern, leading to the greatest DTE observed.

Furthermore, Pang et al. [28], reported increased activation of the default mode network (DMN) during a strong desire to void compared with the empty-bladder state. An increase in the resting state DMN connectivity was also associated with decreased dual-task performance [37]. Usually, the DMN is activated during the resting state, and is strongly inhibited in goal-directed tasks to maintain attention on task performance. Thus, the lack of deactivation of DMN, during both desire to void and dual-tasking, might compete for resources between the DMN and task-related brain regions, reducing the ability to maintain attention on the task or even preventing individuals from creating motor plans with efficiency, resulting in reduced gait control.

Interestingly, our results demonstrate that during the moderate desire-to-void condition, our participants, on average 72.90 ± 5.90 years old, took on average of 12.61 and 13.73 s to complete the TUG test during the cognitive and motor dual-task respectively. Those scores exceed the normative values for their age range, indicating an increased risk of falling during dual-task walking while experiencing a moderate desire to void [38]. It is important to highlight that participants with MoCA scores lower then 26 took longer to perform the TUG test during the cognitive and motor dual-task while experiencing a moderate desire to void. It has already been suggested that interindividual variance in cognition could explain a substantial variance of dual-task performance in older adults [39]. Although it was not the main focus of the present study, based on our results, it is plausible to suggest that older women with changes in cognitive function could be exposed to an increased risk of falling when experiencing a moderate desire to void.

Contrary to our hypothesis, the ICIQ-SF scores did not correlate with lower TUG test scores or higher DTE when performing the dual-task walking while experiencing a moderate desire to void. As we did not use pad tests to quantify leakage volume after each condition, one factor that could explain the lack of association is whether the participants maintained continence while “triple-tasking" [40]. Hence, the lack of such association could be explained by the fact that the participants with higher ICIQ-SF scores might have preferred to allocate cognitive resources to the primary or secondary task instead of controlling continence. Although no correlations between ICIQ-SF score with TUG test performance and DTE were detected, distinct performance operating characteristics were observed comparing participants with continence and those with incontinence.

Understanding the desire to void in a dual-task paradigm is essential in the health of older women. Promising applications of our results include various human performance tasks, such as any daily situation where older women experience a moderate desire to void. Recognizing performance characteristics during those situations allows cognitive overload and better design of coping strategies for women with UI to be considered, consequently preventing falls. Like dual-task training [41], “triple-task” training (dual-task plus desire to void) could reduce brain activity overlap when dual-tasking, presumably decreasing interference. This could help older women to improve isolated tasks (e.g., obstacle crossing, attentional gait, and toilet training or bladder control) or free some attentional resources and probably help them to deal with the increased cognitive load in dual-task situations. Further studies must be conducted to test such a hypothesis.

This study has some limitations. First, the findings cannot be generalized to all older women, as we included participants with varying continence status/severity and cognitive profiles, and our recruitment strategy may have favored healthier and more robust community-dwelling women. Future research is warranted to examine the influence of moderate and severe desire to void on dual-task performance in older adults, considering different UI types and controlling for cognitive function. Second, although the study was powered for the primary within-subject comparison, it may be underpowered to detect interaction effects, which should be interpreted as exploratory. Third, we assessed the desire to void using a subjective scale. This may have induced fear of urine leakage, preventing some participants from achieving a true moderate desire to void, especially those with reduced self-confidence in holding urine [6]. Finally, our protocol induced women to experience a moderate instead of severe desire to void, which might not have caused enough of a sensory load.

Limitations notwithstanding, this work is innovative in that it investigates the desire-to-void effects on the walking ability of older women during dual-tasking. Multifarious and complex walking tasks measure the ability to walk in distinct and challenging environments and may provide a better understanding of the ability to function in daily life than a simple, standardized gait measurement. The dual-task paradigm used in the present study may simulate some of the attentional demands encountered in everyday situations in which individuals experience a moderate desire to void and the need to reach the toilet promptly. Findings from this study indicate that older women with and without UI cannot meet the cognitive demands required to perform walking in everyday life owing to a lack of personal resources and a reduced ability to maintain attention on the walking task. It is plausible that increased distraction whilst attempting dual-task walking and simultaneously maintaining continence may contribute to the association of UI and fall risk in older adults.

## Conclusion

Experiencing a moderate desire to void while walking negatively impacts gait performance in aging women, acting as a source of diverted attention during dual-tasking in older women. Gait performance declines with additional tasks, indicating a cognitive cost from dual-tasking and an added cognitive cost from the full-bladder sensation.

## Supplementary Information

Below is the link to the electronic supplementary material.

Supplementary file1 (PDF 178 KB)

## Data Availability

The datasets generated and/or analysed during the current study are not publicly available but are available from the corresponding author on reasonable request.

## Declarations

### Prior Presentation

Partial and preliminary findings from this study were presented at the International Continence Society (ICS) Annual Meeting 2023 (Toronto, Canada). The present manuscript includes the complete and updated analyses.
