# Supplementary material for: Bladder Sensation and Gait: A Cross-Sectional Observational Study of Dual-Task Interference in Older Women
Source: Int Urogynecol J. 2026 Mar 30;37(8):2605–14. doi: 10.1007/s00192-026-06573-z (PMC13486069; doi:10.1007/s00192-026-06573-z)
Supplement: Supplementary file 1 [file 192_2026_6573_MOESM1_ESM.pdf]

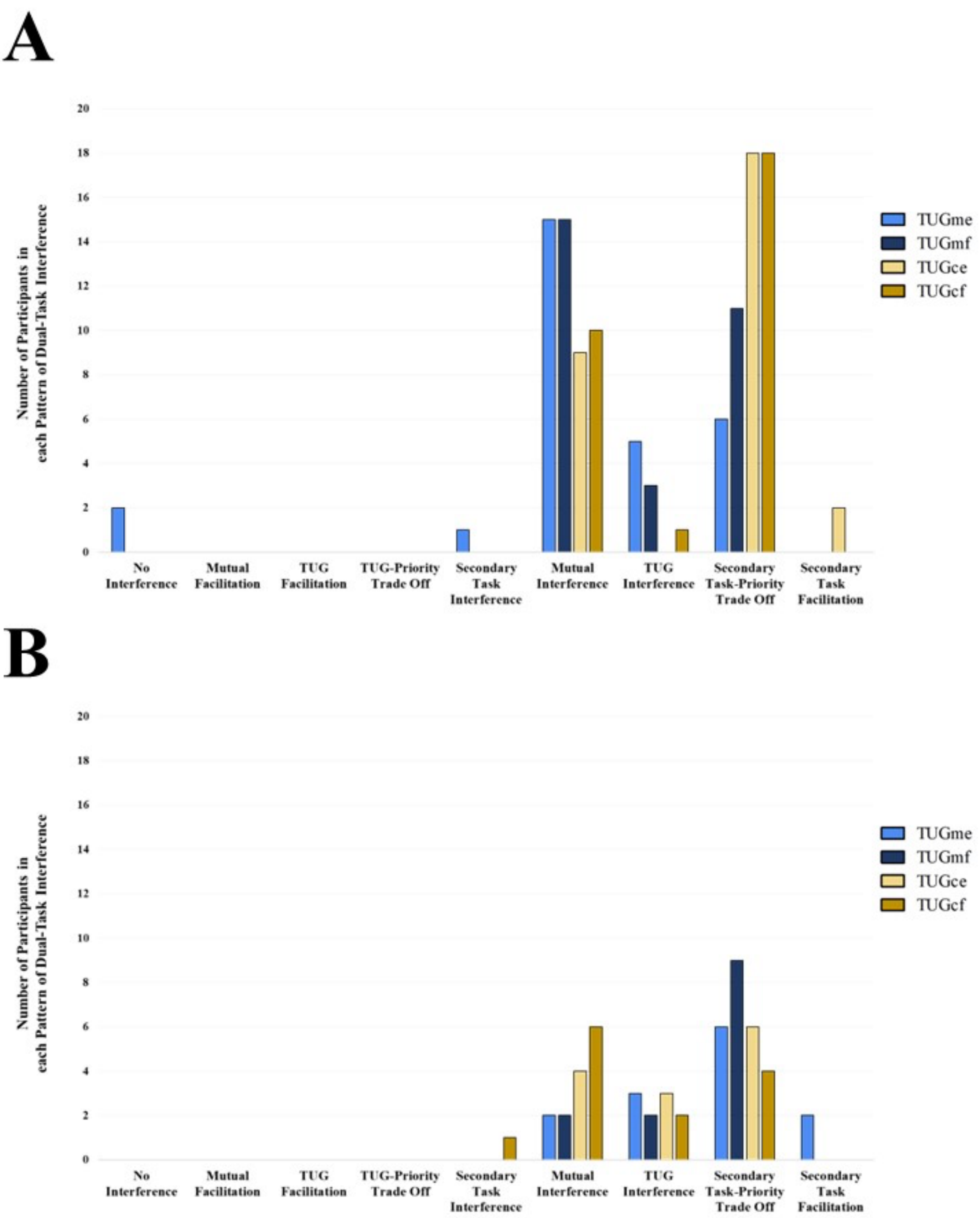

Supplementary Figure 1. Number of participants in each patters of dual-task interference. **(A)** Participants with ICIQ Scores ≥ 1 (i.e., participants with slight, moderate and severe incontinence); **(B)** Participants with ICIQ Scores = 0 (i.e., continent participants with no signs of incontinence).
